# Unravelling the complexities of end-of-life critical care interventions: what drives medico-legal complaints to physicians?

**DOI:** 10.1186/s12913-025-13968-z

**Published:** 2026-01-07

**Authors:** Marc-André Blier, Rana Aslanova, Karen Lemay, Shan Jin, Teena Levesque, Gary E. Garber

**Affiliations:** 1 Dalla Lana School of Public Health, University of Toronto, Health Science Bldg, 155, Toronto, ON M5T3M7 Canada; 2 Department of Safe Medical Care Research, Canadian Medical Protective Association (CMPA), Ottawa, ON Canada; 3 Faculty of Medicine, Department of Medicine and the School of Epidemiology and Public Health, University of Ottawa, Ottawa, ON Canada

**Keywords:** End-of-life care, Critical care medicine, Medico-legal, Advance directives, Shared decision making and communication

## Abstract

**Background:**

This study sought to describe the key medico-legal issues associated with end-of-life critical care interventions in Canada. We explored the most common themes of a disagreement between patients or substitute decision makers (SDM) and treating physicians related to intervention decisions, and complaints against physicians, that were identified and criticised by peer experts as contributing to medico-legal risk.

**Methods:**

A national repository was retrospectively searched for cases involving end-of-life care that were supported by the Canadian Medical Protective Association (CMPA) between 2018 and 2022. The study involved patients admitted to surgical and medical intensive and critical care units, emergency departments and residents in long term care settings. The frequencies and proportions of the top risk factors leading to medico-legal risks and the most frequent allegations were calculated by exploring factors that contributed to each incident.

**Results:**

We identified 93 eligible cases, involving 142 physicians. Family medicine was the most frequent specialty named in 32% of the cases. The median age of 93 unique patients was 78 years. Common reasons for complaints were communication breakdown between a physician and a patient/SDM and deficient assessment. The most frequent contributing factors criticised by peer experts were communication breakdown between a physician and a patient/SDM and inadequate documentation.

**Conclusions:**

The most common omission identified by peer experts involved a physician’s communication with a patient/SDM. Physicians may reduce their medico-legal risk by exploring effective techniques for optimal communication to improve understanding in end-of-life discussions, with the goal of high-quality patient care.

**Supplementary Information:**

The online version contains supplementary material available at 10.1186/s12913-025-13968-z.

## Background

Intensive, or critical, care units (ICU/CCU) in Canada are a vital component of acute care, where critically ill patients receive advanced life support therapies aiming to increase their survival and enhance their quality of life [1]. However, life-sustaining interventions may not always align with patients’ values, preferences and clinical indications [2]. Recent studies have demonstrated that early integration of palliative care into ICU management, enhances patients satisfaction, alleviates physical symptoms, and augments quality of their life [3, 4]. 

ICU/CCUs are a costly resource because they require high staff-to-patient ratios for intensive patient monitoring, and complex and potentially interactive treatments, involving multiple interventions delivered by different healthcare professionals [5, 6]. The frequency of critical care usage is increasing year over year. In 2023-24, there were just over 4,800 ICU beds in Canada [7], up from approximately 4,500 in 2018 − 19 [7]. Age-stratified analyses have demonstrated that ICU admission and mechanical ventilation rates changed variably by age, with increasing rates primarily among people under the age of 50 [8]. Studies suggest that population growth, increases in severity and complexity of critical illness, and Canada’s overall ageing population will likely all contribute to ICU/CCUs’ challenges in meeting future demand, and raising concerns for an appropriate allocation of resources [1, 9–12]. 

For some patients in an acute health decline, the ICU/CCU can be a part of appropriate end-of-life care, offering services that are specific to patient comfort and needs, while respecting their desires and preserving their dignity [13, 14]. End-of-life (EOL), or comfort care, whether it is related to a terminal illness or age-related physiological deterioration, presents many challenges (e.g., management of pain, alleviation of suffering or optimization of quality of life prior to a patient’s death) for physicians, as well as for patients and their families [15]. Critically ill patients at their EOL should have shared discussions about their preferences with their care team and family, ensuring that advance directives and patient-centred goals of care are understood [16]. These discussions enable both a multidisciplinary care team and family to advocate for the patient’s wishes when the patient has lost ability to make decisions or communicate their preferences [17]. 

Despite having predictors of poor outcomes or goals inconsistent with life support, some patients continue to be admitted to critical care settings. The published literature also delineates non-beneficial and inappropriate treatments that were not in the patient or substitute decision maker (SDM) wishes, that nevertheless may be offered by physicians to appease family wishes and expectations (e.g., enduring invasive ventilation) [18–21]. A certain level of these interventions are inevitable during very distressing times in ICU settings. Multidisciplinary critical care teams frequently have to deal with uncertainty of prognosis and outcomes of life-threatening conditions. They have to simultaneously react to changing physiology of the patient with resuscitative measures while considering palliative interventions, and must communicate rapidly-changing situations to patients and families [22]. The extent, variation and justification of critical care interventions may also be influenced by the physician’s concerns about complaints from patients or SDMs [23]. The risk of potential conflict intimidates some physicians, and contributes to their offer of non-beneficial therapy, as acquiescing to requests is often deemed easier than discussing the therapy’s non-beneficial nature [22, 23]. 

We aimed to explore the modifiable factors contributing to physicians’ medico-legal risks, the common themes of advance therapies that were not offered, and the themes of disagreements between patients or SDMs and treating physician(s). This study addressed the gap in the published literature of healthcare-related risk factors associated with end-of-life critical care interventions from Canadian medico-legal cases.

## Methods

We performed a descriptive study and content analysis of medico-legal cases supported by the Canadian Medical Protective Association (CMPA) that were closed between January 1, 2018 and December 31, 2022.

The CMPA is a national, membership-based, not-for-profit mutual defence organization that offers medico-legal support, advice, and education to physicians. At the time of this study, the CMPA represented over 113,000 members (estimated at over 95% of physicians in Canada). The Association maintains a large national repository that includes information on advice calls to the Association, legal actions, and complaints to regulatory authorities (Colleges) and hospitals. Physician members contact the CMPA when seeking advice or support for medico-legal matters.

The concepts and themes of this paper arose out of team discussions of the de-identified clinical summaries and expert opinions in the selected medico-legal cases. The study is reported according to the ICMJE Recommendations for the Conduct, Reporting, Editing, and Publication of Scholarly Work in Medical Journal and the Reporting of Studies Conducted using Observational Routinely-collected Data (RECORD) reporting Guidelines [24, 25]. 

### Data sources

This study used routinely collected and coded medico-legal data from the CMPA repository. Each closed medico-legal case in our sample is based on civil legal actions or complaints against a physician to a hospital or to provincial or territorial medical regulatory authority (College) (Appendix A). Allegations reflect the patient, family or SDM’s perception of the problem(s) that occurred during care. As part of the medico-legal process, a case may be reviewed by peer experts. These opinions are used by CMPA to identify contributing factors related to the medico-legal risk. Peer experts refer to physicians who interpret and provide their opinion on clinical, scientific or technical issues surrounding the care provided. They are typically of similar training and experience as the physicians whose care they are reviewing. Peer experts may not be critical of the care reflected in the allegation or may have criticisms that are not part of the allegations. Peer experts are not advocates for either party but rather provide an impartial and credible professional opinion about whether the standard of care was met. Coding of peer expert opinion is guided by the CMPA’s established Contributing Factors Framework (CFF) [26]. After case closure, de-identified medico-legal records are routinely coded by medical analysts, who are experienced and trained registered nurses. Clinical information is coded using the Canadian Enhancement to the International Statistical Classification of Diseases and Related Health Problems (ICD-10-CA) [27], and the Canadian Classification of Health Interventions (CCI) Code of a Critical Care Intervention [28]. Quality assurance occurs on a regular basis to assure data accuracy and reduce misclassification of case codes. There are various procedures in place to ensure the quality and consistency of the coding, including independent case reviews; electronic flagging of inconsistent values; and group review in the form of audits. The study case examples were purposely high-level to prevent identification of both patients and healthcare providers while still providing opportunity for physicians to relate the examples to their specific clinical practice.

### Participants and case selection

**Fig. 1:**
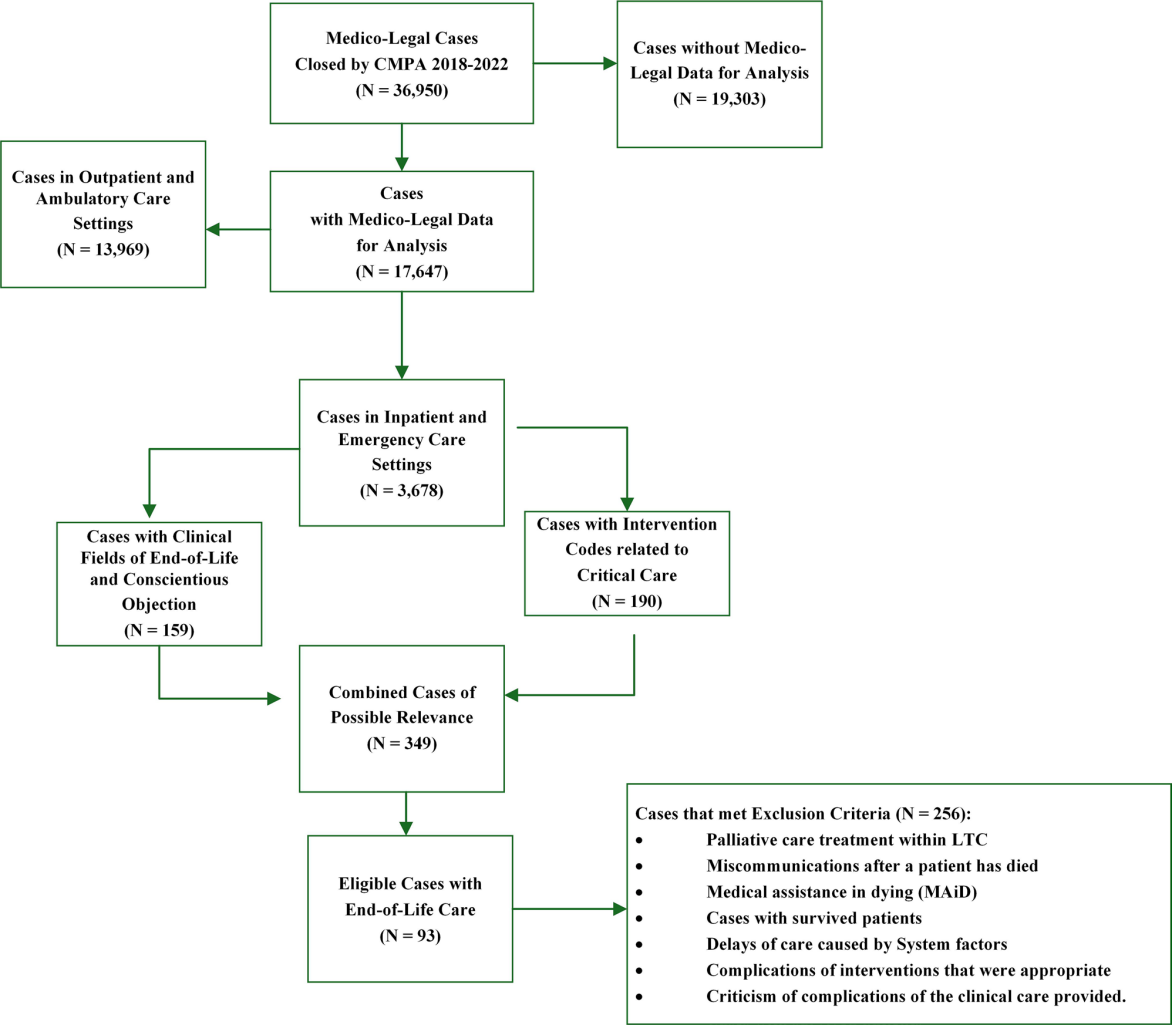
Diagram showing case eligibility and inclusion

The study population consisted of inpatients with an end-of-life event, including those in long term care settings and emergency departments. Case problem types were stratified by civil legal actions, College and hospital complaints. The eligibility of the cases was identified by applying the following inclusion criteria: patient aged 18 years or older; cases that were closed between 2018 and 2022 and involving physicians of all specialties; and cases with the CCI code for critical care intervention. Cases were excluded if they met exclusion criteria by one of the parameters presented in Fig. 1.

### Variables

SAS was used to abstract variables of interest: case type; date of clinical encounter; patient demographics (age and self-reported gender); settings of critical care; patient diagnoses, complications and outcomes; reason for complaint and associated contributing factors; intervention decision; physician specialty.

### Statistical methods

For variables of interest, we calculated frequencies and proportions and used univariate statistics. The categorical variables were described as proportions and continuous variables were presented as median and interquartile range (IQR). Eligible cases were grouped thematically based on the allegation and corresponding contributing factor by employing the CMPA’s Contributing Factors Framework (CFF) [26] designed to identify factors contributing to patient safety incidents. The CFF was used to identify data themes, and a sample of individual cases was reviewed to identify illustrative examples. To protect the privacy of both physicians and patients, any category that summed to fewer than 10 was censored. We used SAS version 9.4 (SAS Institute Inc., Cary, NC) and Microsoft Excel 365 (Microsoft Corporation. Redmond, WA).

## Results

The CMPA closed 36,950 medico-legal cases between 2018 and 2022. Based on the case extraction criteria and manual review of individual case clinical summaries, a total of 93 cases met eligibility criteria, including 66 (71%) College, 14 (15%) hospital complaints, and 13 (14%) civil legal matters. Clinical encounters in these cases occurred between the years 2000 and 2022, as the time from a case occurrence to a file closure may take from a few months to several years. These cases involved 93 unique patients with the median age of 78 years (IQR 22.1–96.0). More patient characteristics are presented in Table 1.

There were 142 physicians involved in the 93 cases, as a case can involve more than one physician or physician specialty. The most frequently involved specialty was family medicine (32%, 45/142), followed by 15% (22/142) of internal medicine specialists. Distribution of physician specialties is presented in Appendix B (Table B1).

The top three complaints from a patient/family/SDM (Appendix C, Table C1) were communication breakdown between a physician and a patient/SDM, deficient assessment, and the physician’s unprofessional manner. These allegations, with the examples from the medico-legal cases, are presented in Table 2.

**Table 1:** – Patients demographics and care settings in the 93 cases

Characteristics	*N* (%) of patients
**Demographics **(*N* = 93)^a^	
** Age range (year)**:	
18–29	˂10 (< 10)
30–64	19 (20.4)
65–79	26 (28.0)
≥ 80	41 (44.0)
** Gender**	
Male	56 (60.0)
Female	37 (40.0)
** Location of Inpatient Care Settings**	
ICU/CCU ^b^	29 (31.2)
Palliative Care	<10 (< 10)
Emergency	16 (17.2)
Ward	46 (49.5)
Operating Room	<10 (< 10)

ªBased on 93 patients with known age and self-reported gender

^b^ICU – Intensive Care Unit, CCU – Critical Care Unit

**Table 2:** – Summary of examples of the most common patient/SDM’s allegations from the 93 medico-legal cases

Allegations, *N* (%) of Cases	Examples
1. Communication breakdown between a physician and a patient/SDM^a^, 46 (48.9)	A physician:
	- Failed to speak to a patient directly to obtain their resuscitation status. - Did not include a patient/SDM in level of care discussions. - Wrote a “Do Not Resuscitate” (DNR) order without first having a discussion with a patient’s SDM. - Failed to inform a family of the patient’s admission to the intensive care unit (ICU). - Failed to discuss the decision to implement palliative care with the patient or explain what types of care were included in palliative care. - Involved the Consent and Capacity Board (CCB) to determine if an SDM’s decision was within the patient’s best interests.
2. Deficient assessment of a patient, 31 (33.0)	A physician:
	- Performed an inadequate assessment and reassessment of a patient’s condition. - failed to diagnose a patient’s condition in a timely manner. - Failed to immediately recognize the severity of the patient’s condition.
3. Unprofessional manner, 29 (31.0)	When:
	- An SDM felt coerced or bullied into changing the level of care for their parent. - A physician inadequately managed the privacy and care of a patient. - A physician communicated in a harsh manner and failed to show compassion and empathy.

^a^Communication issue with a patient, patient’s family, or substitute decision maker on the part of the healthcare provider, not the patient. This includes ignoring/dismissing patient concerns

In 52.7% (49/93) of the cases, the allegations were related to non-offers of critical care interventions. The themes of non-offered interventions are displayed in Appendix D (Table D1). Clinical examples of non-offers from the peer expert opinion are presented in Table 3. We found 37 (39.8%) cases involving allegations and disagreements with family or SDM relating to discontinuation of life sustaining therapies, and focusing interventions on palliative and comfort care. The themes of disagreement included: changes in goals of care, changes to clinical management, lack of appropriate diagnostic investigations prior to the designation of a palliative status, lack of consent to discontinue life-sustaining therapy, and cancelling a specialist consultation. Case examples with peer expert criticism are presented in Appendix E (Table E1). To identify factors contributing to the medico-legal risk involving care management at the end of a patient’s life that resulted in allegations, the analysis was focused on 47% (44/93) of the cases with peer expert criticism (Appendix F, Tables F1/F2). In the remaining 53% of the cases, peer experts supported the clinical care provided by the implicate physician(s). In the cases critiqued by peer experts, the most prominent contributing risk factors were communication breakdown with a patient or SDM and inadequate documentation. Summarized examples of these risk factors are reported in Table 4.

**Table 3:** – Case examples of non-offers of care interventions at the end-of-life in 49 (52%) medico-legal cases with a patient/SDM allegations and expert opinions

Interventions Not Offered	Case Examples
Cardiopulmonary Resuscitation (CPR)	*Allegation*: an SDM agreed that cardiopulmonary resuscitation (CPR) would be harmful but questioned why the patient was not offered other treatment options. *Clinical details*: there was a moral distress among ICU staff regarding medical management of a patient. The critical care team felt it inappropriate to pursue CPR or re-intubation. After a meeting with the SDM, a physician wrote the DNR (do not resuscitate) order. *Expert opinion*: a peer expert opined the DNR decision was necessary and appropriate but was critical of a physician for failing to document a contemporaneous note outlining the details of the DNR discussion.
Pharmacotherapy	*Allegation*: an SDM alleged inappropriate use of pharmacotherapy and deficient monitoring of a patient’s respiratory status *Clinical details*: a post-operative patient developed somnolence and respiratory depression. Because of a pre-existing “DNR” order, the patient was not intubated. There was also a delay in initiating continuous positive airway pressure (CPAP) and the patient’s respiratory distress progressed to cardiac arrest. *Expert opinion*: a peer expert opined that a more detailed scope of treatment discussion would have added clarity, and the physician should have recommended intubation and recognized that this treatment was inconsistent with patient’s directive.
Admission to an ICU	*Allegation*: a patient’s SDM alleged a patient was denied admission to an intensive care unit. *Clinical details*: a physician discussed with a patient and their SDM that intensive care admission would be unlikely to allow the patient to survive. The patient did not want to be transferred to ICU and comfort measures became the clinical focus. *Expert opinion*: a peer expert found the care provided was reasonable and delivered with compassion.

**Table 4:** – Summary of case examples of the most common contributing risk factors from the 44 (47%) medico-legal cases with peer expert criticism (2018–2022)

Contributing Factors, *N* (%) of Cases	Case Examples
I. Communication breakdown with a patient/SDM, 26 (28.0)	
- Family expectations regarding severity of a patient’s condition	A breakdown in communication occurred between a physician and a patient’s SDM when the family was still hopeful that the patient would recover despite being given a terminal diagnosis, and the involvement of palliative care. A breakdown in communication occurred when a patient’s diagnosis and prognosis had not been explained to the family or they did not understand the explanations. **A physician** did not inform a SDM of a patient’s urgent medical condition.
- Goals of care and resuscitation status discussions	A simple “DNR” order was too ambiguous; a more detailed scope of treatment discussion to clarify the patient/family expectations would have prevented a patient’s death. At a meeting with a patient’s spouse, a physician did not clearly explain that the patient was near the end-of-life. A family was unaware that a level 3 status did not include resuscitation in the event of cardiac arrest.
- Discontinuation of critical care or life sustaining interventions	A patient’s SDM did not perceive their conversations with a physician as compassionate or caring when the discussion involved the financial cost of critical care and the allocation of nursing resources. College stated their expectation that physicians always conduct discussions about withdrawal of life support in a sensitive manner. A patient’s family was upset when the patient was moved to a palliative care bed prior to discussion.
- Clinical care management options	A physician did not discuss the risks and benefits of anticoagulation therapy with SDM.
II. Documentation deficiencies, 21 (22.0)	Non-existent or poorly detailed notes about: - rationale for clinical decision-making - a patient’s status including prognosis and clinical progress. - the clinical management plans. - results of diagnostic investigations. - details of crucial discussions with patients and their SDMs - patient’s verbalized end-of-life wishes.

## Discussion

The study identified 93 cases of medico-legal complaint relating to end-of-life decisions. During a detailed review, communication breakdowns was the most contributing factor, which was among the reasons for complaint in 49% of cases, and was criticised by peer experts in 28% of cases. Communication issues were most often associated with different expectations of a patient’s near-to-death outcomes by a family/SDM and the physician, due to breakdowns in communication or misunderstanding about the patient’s medical condition. Studies on physician-patient communication have identified patients’ concerns even when their physicians considered the communication adequate or even excellent [29–31]. Meetings with a family/SDM may be undertaken when a patient is unconscious, intubated, sedated, in significant pain, sharply deteriorating, or in a state of cognitive incompetence [32]. Research has identified that conflicts, disagreements, misunderstandings, and different expectations are common in critical care settings, due to communication that occurs in these stressful situations [33, 34]. 

Our analysis of complaints also identified cases where families felt that the physician did not clearly communicate the goals of care, treatment options, or changes in clinical management or generally communicated in an insensitive manner. This finding aligns with several studies in the literature, including a study by Azoulay et al. that showed that half of family representatives of ICU/CCU patients failed to comprehend the diagnosis, prognosis, or treatment of the patient because of inadequate communication with a physician [35]. A qualitative study that surveyed patients and their relatives revealed that a lack of sensitivity and poor communication with the patient and their family after a patient safety event influenced their decision to take legal actions [36]. Virshup et al. concluded that most complaints against physicians are generated out of anger, due to lack of communication or failure to develop rapport, rather than deficiency of the physician’s skill [37]. Physicians and clinical care teams in the ICU/CCU should consider the importance of clear, sensitive communication as an essential component of the physician-patient relationship and to reduce the risk of medico-legal complaint. Structured communication tools, such as the SPIKES model [38], may help facilitate clear communication of difficult news to patients and family members, emphasizing the need for an appropriate setting, clarity in describing the disease process, opportunities for patients and SDMs to ask questions and receive answers, and a summary of the treatment plan for the patient and care team. This may also assist with concerns about professionalism that we saw in some medico-legal cases, as more time is allowed for SDMs to communicate in family meetings which is known to increase family satisfaction and decrease conflict between SDMs and the care team [33]. 

Other members of the clinical care team can also offer insights and identify when the treatment plan may not align with the patient’s values or to help communicate with patient/SDMs. Research suggests that nurses and respiratory therapists often identify patients with incongruent resuscitation status and can alert the physician to this [39]. Social workers also provide communication during and outside formalized family meetings. Clinical guidelines may provide useful frameworks, describing the way that all members of the clinical care team can support clear communication of care plans before, during and after family meetings [40]. 

Half of the cases assessed in this study were related to non-offers of care, the most common interventions that were not offered to patients were refusal of critical care life-sustaining interventions and changes of care settings. In the authors’ jurisdiction, Ontario, physicians must engage with patients or SDMs during treatment planning [41]. A treatment plan is a proposed package of therapies presented to the patient or SDMs based on what is medically feasible and in concordance with the patient’s expressed wishes and values, that can be accepted or rejected, the later leading to conflict resolution processes [41]. Within treatment plans there may be non-offers: interventions not offered due to non-beneficial status. The decision to make an intervention non-offer hinges on the belief that the intervention is non-beneficial. While the term non-beneficial does not have consensus there have been attempts to clarify its meaning with the most commonly held meaning of the term being, “advanced curative/life-prolonging treatments that are not consistent with the goals of care (as indicated by the patient) [39]. The prognostic certainty may be immediately clear to the physician as in the non-beneficial status of CPR in cardiac arrest from terminal, irreversible disease [42]. Other times this estimation is not as obvious but falls on the physician’s gestalt of the situation which may include comorbid disease and function status (e.g. clinical frailty scoring [43]) weighed against harms incurred by the intervention [44]. Of note, while advanced age is associated with lower ICU survival it is insufficient in isolation to determine suitability for advanced therapies [43]. Non-offers are a necessary component of treatment planning but where genuine uncertainty from the physician exists regarding appropriateness of an intervention, a time limited trials of therapy may be offered [45]. Time limited trials involve an intervention for a defined period of time before re-evaluation of clinical endpoint. For example, trial of intubation for five days with extubation on the final day as not to prolong ventilation. Time limited trials increase communication with families as regular dialogue is required, reduce length of stay, and increase consensus in decision making [46]. 

Other relevant risk factors that were identified in this study, such as communication breakdown between physicians or between a physician and another healthcare provider, can be investigated in future research. Finer details of exploring non-beneficial therapy, prognostication, and bias in decision making are important considerations for future study.

### Study limitations

The retrospective nature of medico-legal cases identifies association rather than causation, and is influenced by recall, hindsight and outcome biases. Patients who experience healthcare related harm or their SDMs may not file a complaint. As well, physicians seek CMPA assistance at their own discretion and may not seek support with all complaints. Therefore, this study may not represent a comprehensive analysis of all Canadian medico-legal cases on the topic of interest.

## Conclusions

This study provides insight into the frequency and factors contributing to complaints associated with critical care intervention decisions at the end of a patient’s life. The leading cause of both patient/SDM allegation and contributing factor being communication breakdown. Physicians and hospitals can focus on this area to reduce risk of allegation through additional training in communication while enhancing patient/SDM satisfaction and advancing the delivery of critical care services to patients at the end of their life.

## Supplementary Information

Below is the link to the electronic supplementary material.

Supplementary Material 1

## Data Availability

All data generated and analysed during this study are included in this published article and its *Supplementary Information* file. More granular data could not be released due to privacy and ethical restrictions.

## Abbreviations

CMPA: The Canadian Medical Protective Association
EOL: End-of-life
SDM: Substitute decision maker
ICU/CCU: Intensive care unit/Critical care unit
ICMJE: International Committee of Medical Journal Editors
RECORD: REporting of studies Conducted using Observational Routinely-collected Data
CFF: Contributing Factors Framework
ICD-10: International Statistical Classification of Diseases and Related Health Problems 10th Revision (2019)
CCI: The Canadian Classification of Health Interventions
SAS: Statistical software
IQR: Interquartile range
DNR: Do Not Resuscitate
CPR: Cardiopulmonary Resuscitation
CPAP: Continuous Positive Airway Pressure
