# Supplementary material for: Unravelling the complexities of end-of-life critical care interventions: what drives medico-legal complaints to physicians?
Source: BMC Health Serv Res. 2026 Jan 7;26:174. doi: 10.1186/s12913-025-13968-z (PMC12869921; doi:10.1186/s12913-025-13968-z)
Supplement: Supplementary file 1 — Supplementary Material 1 [file 12913_2025_13968_MOESM1_ESM.docx]

**Supplementary Information**

Appendix A: Definition of medico-legal cases by problem type

Appendix B: Distribution of physician specialties (Table B1)

Appendix C: Patient/family/SDM reasons for allegation(s) (Table C1)

Appendix D: Non-offers of ICU/CCU interventions with case examples (Table D1)

Appendix E: Case examples of discontinuation of life sustaining therapies (Table E1)

Appendix F: Contributing risk factors (Table F1) and their definitions (Table F2)

**Appendix A: Definition of medico-legal cases by problem type**

In the current study, each closed case represented a civil legal, College, or hospital matter

defined as follows:

- *Civil legal action:* A physician was served or received a claim from the plaintiff or third

party claimant or a defense was filed on their behalf.

- *Civil legal threat:* The physician has received a communication suggesting that a civil legal action will be initiated against the physician.
- *College complaint:* A complaint was lodged against a physician to a medical licensing

(regulatory) authority.

- *College disciplinary matter:* The matter was considered by a committee of a medical

licensing (regulatory) authority whose function it was to discipline.

- *College preliminary matter*: An investigation, peer assessment, professional inspection,

or request for personal information was commenced by a medical licensing (regulatory)

authority. (These matters were distinct from those undertaken by a fitness committee,

complaints committee, or discipline committee).

- *Hospital complaint:* A complaint lodged against a physician to a hospital or health

authority.

If multiple case types reflected the same physician complaint, then we included only the most

serious case type (legal > College > hospital, in order of decreasing severity).

**Appendix B:**

**Table B1** – Distribution of physician specialties in the 93 cases (n=142)

| **Physician Specialty** | **N (%) of physicians** |
| --- | --- |
| Family medicine or General practice | 45 (31.5) |
| Internal medicine | 22 (15.4) |
| Emergency medicine | 18 (12.6) |
| Critical care medicine | 13 (9.1) |
| General surgery | 8 (5.6) |

**Appendix C:**

**Table C1** – A patient/family/SDM’s reason for allegations in the 93 medico-legal cases

| **Allegations** | **N (%) of cases ^a^** |
| --- | --- |
| Communication breakdown between a physician and a patient/ SDM | 46 (49.5) |
| Deficient assessment | 31 (33.3) |
| Unprofessional manner | 29 (31.2) |
| Poor decision-making regarding management | 22 (23.7) |
| Failure to perform a diagnostic test or intervention | 20 (21.5) |
| Inadequate consent process | 18 (19.4) |
| Diagnostic error | 16 (17.2) |
| Choice of medication not optimal | 15 (16.1) |
| Inadequate patient monitoring or follow-up | 13 (14.0) |
| Inappropriate/failure to transfer | 13 (14.0) |

**Appendix D:**

**Table D1 –** Non-offers of ICU/CCU interventions in the study case sample

| **Type of Care Intervention** | **Examples** | **Number of cases** |
| --- | --- | --- |
| Critical care interventions | - Endotracheal intubation - Non-invasive and invasive ventilation - Cardiopulmonary resuscitation - Defibrillation - Dialysis | 19 |
| Change of care settings | - Non-tertiary care to tertiary care - Long term care to hospital - Transfer to ICU | 16 |
| Pharmacotherapy | - Pain management - Sedation | 7 |
| Specialist consultation | - Palliative care team - Cardiology - Critical care intensivist | 6 |
| Diagnostic investigation | - Computerized tomography | 5 |

**Appendix E:**

**Table E1** – Summary of case examples with a family/SDM allegations of discontinuation

of life sustaining therapies criticized by peer experts in 37 (40%) cases.

| **Discontinuation of Life Sustaining Therapies** | **Case Examples** |
| --- | --- |
| **Withdrawn of life sustaining support without consent** | ***Allegation:*** a physician did not communicate with an SDM regarding important decisions to be made.  ***Clinical details:*** a patient with a life-threatening condition was intubated. After consultation with a specialist, it was determined the patient was not a candidate for surgery. Attempts to contact the patient’s family/ were unsuccessful. When the patient’s condition deteriorated, the physician extubated the patient and the patient passed away.  ***Expert opinion:*** life sustaining support should not have been withdrawn without consent. |
| **Rapid discontinuation of life sustaining treatment** | ***Allegation:*** a patient’s sibling alleged a physician made the decision to discontinue patient's treatment too quickly.  ***Clinical details:*** a physician and an ICU nurse separately had detailed discussions with a patient about the consequences of discontinuing life sustaining treatment and determined the patient was competent when the decision to stop treatment was made. The critical care team respected the patient’s wishes including not informing the patient’s family of the decision.  ***Expert opinion:*** The peer expert opined the physician’s decision-making process was appropriate and the care provided exceeded the standard of care. |

**Appendix F:**

**Table F1** – Top 10 contributing risk factors in the 44 cases criticized by peer experts

| **Contributing Factors** | **N (%) of Cases** |
| --- | --- |
| Communication breakdown between a physician and a patient/family/SDM | 26 (27.8) |
| Inadequate documentation | 21 (23.7) |
| Deficient assessment | 11 (12.4) |
| Knowledge issues regarding practice management | 10 (10.3) |
| Communication breakdown between physicians | <10 (<10) |
| Failure to perform a diagnostic test or intervention | <10 (<10) |
| Communication breakdown between a physician and another healthcare provider | <10 (<10) |
| Insufficient knowledge/skill | <10 (<10) |
| Failure to refer | <10 (<10) |
| Choice of medication not optimal | <10 (<10) |

**Table F2** – The CMPA Definitions of the Contributing Factors (CF)

| **Contributing Factors** | **CMPA Definition** |
| --- | --- |
| Choice of medication suboptimal | Decision making related to the choice of medication, such as a suboptimal dose or route or stopping medication. Includes amount, frequency, quantity, or indication for medication |
| Communication breakdown between a physician and a patient/ SDM | Communication issue with a patient, patient's family, *or substitute decision maker* on the part of the healthcare provider, not the patient  Note: Includes ignoring/dismissing patient concerns. If related to physician communication style or behavior consider unprofessional manner |
| Communication breakdown between physicians | Communication issues between physicians involved in a patient’s care. Includes ignoring/dismissing another physician’s concern. If related to physician communication style or behavior, consider unprofessional manner. |
| Communication breakdown between a physician and another healthcare provider | Communication issues between a physician and other healthcare provider involved in a patient’s care. Includes ignoring/dismissing healthcare providers concerns. If related to physician communication style or behavior, consider unprofessional manner. |
| Deficient assessment | Deficient histories and general evaluation. Includes deficient evaluation for the purpose of prescribing or related to the performance of a procedure. |
| Diagnostic error | Misdiagnosis, failure (missed diagnosis) or delay to establish an accurate and timely explanation of the patient's health problems (diagnosis) or communicate that explanation to the patient. |
| Failure to perform a diagnostic test/intervention | Delay or failure to perform a diagnostic test (excludes assessment) or therapeutic intervention (e.g., ECG, pharmacotherapy, surgery). |
| Failure to refer | Delay or failure to refer a patient or consult another physician |
| Inadequate consent process | Issues involving the discussion or documentation of the risks, limitations, side effects or alternative options of a diagnostic test or therapeutic intervention, e.g. pharmacotherapy, surgery. Includes consent for physical assessment or withdrawal of consent. |
| Inadequate documentation | Inadequate, delay or failure to complete documentation (written or electronic). |
| Inadequate monitoring or follow up | Delay, absent or incomplete follow-up of diagnostic test results and/or monitoring of a patient's clinical condition (e.g., vital signs, O2 sats, neuro assessment), and if required act upon abnormal findings and plan future care. |
| Inappropriate/failure to transfer | Delay, failure or inappropriate transfer of a patient between locations, including within the same facility and between facilities |
| Insufficient knowledge/skill | Issues involving a provider's clinical knowledge, skill, technique, training or education. Also includes when the provider is required to complete training or education related to these topics or when a member proactively initiates training or education for the same. |
| Knowledge issues regarding practice management | Issues involving a provider's knowledge regarding practice management including documentation, communication, and professionalism. |
| Poor decision-making regarding management | Issues involving a provider's decision making related to selection and management of patient care, includes treatments not indicated for the patient. |
| Unprofessional manner | Provider manner issue, including style and mode of communication, disruptive, inappropriate or unprofessional behavior. |
